# Peripheral Arterial Tonometry to Measure the Effects of Vardenafil on Sympathetic Tone in Men with Lifelong Premature Ejaculation

**DOI:** 10.1155/2013/394934

**Published:** 2013-03-27

**Authors:** Davide Francomano, Lorenzo M. Donini, Andrea Lenzi, Antonio Aversa

**Affiliations:** Department of Experimental Medicine, Section of Medical Pathophysiology, viale Regina Elena 324, Sapienza University of Rome, 00161 Rome, Italy

## Abstract

To elucidate whether adrenergic overtone is involved in the pathophysiology of men with lifelong (LL) premature ejaculation (PE), we investigated differences in reactive hyperemia index (RHI) responses by using peripheral arterial tonometry (PAT). 20 men with LL-PE (18–40 years) were enrolled in an 8-week, double-blind, placebo-controlled, crossover study and compared with 10 age-matched controls without LL-PE. Primary endpoints were PAT modifications induced by vardenafil 10 mg on demand. Secondary endpoints were the improvement in intravaginal ejaculatory latency time (IELT) as measured by the stopwatch technique and variations in anxiety scores at Stai-X1 for state-anxiety and Stai-X2 for trait-anxiety. At baseline, men with LL-PE showed higher RHI variation (*P* < 0.001), Stai-X1 and Stai X2 scores (*P* < 0.0001, resp.), and prolactin levels (*P* < 0.05) compared with controls. Vardenafil treatment markedly reduced RHI variation in men with LL-PE (*P* < 0.01) when compared with placebo. Mean changes in geometric IELT were higher after taking vardenafil (0.6 ± 0.3 versus 4.5 ± 1.1 min, *P* < 0.01) when compared with placebo. STAI-X1 and STAI-X2 scores fell within the normal range after treatment with vardenafil (*P* < 0.01). Vardenafil was an effective treatment in men with LL-PE; improvements of IELT may be due to increased NO production which is able to reduce adrenergic overactivity and anxiety levels.

## 1. Introduction

Lifelong premature ejaculation (LL-PE) is defined as a “male sexual dysfunction” characterized by ejaculation which always or nearly always occurs before or within about one minute of vaginal penetration and the inability to delay ejaculation on all or nearly all vaginal penetrations and negative personal consequences, such as distress, bother, frustration, and/or the avoidance of sexual intimacy [1]. The organs involved in the emission phase comprise the epididymis, vas deferens, seminal vesicles, prostate gland, prostatic urethra, and bladder neck. The organs participating in the expulsion phase include the bladder neck and urethra, as well as the pelvic striated muscles. The central ejaculatory neural circuit comprises spinal and cerebral areas that form a highly interconnected network. The sympathetic, parasympathetic, and somatic spinal centers, under the influence of sensory genital and cerebral stimuli integrated and processed at the spinal cord level, act in synergy to command physiologic events occurring during ejaculation. A wide number of neurotransmitters, including serotonin (5-HT), dopamine, oxytocin, gamma-aminobutyric acid (GABA), adrenaline, acetylcholine, and nitric oxide (NO), have been shown to be involved in the regulation of ejaculation. Since 1984, the nonselective alpha-blocker phenoxybenzamine was demonstrated to be able to improve premature ejaculation in humans [2]. Subsequent animal studies demonstrated that the effect of selective alpha blockade is obtained by inhibiting the contractile response of the rat seminal vesicle to electrical nerve stimulation [3]. Further evidence indicates that the contractility of the human seminal vesicle is under the control of the NO-cGMP pathway, thus giving a rationale for the use of NO donors in the pharmacotherapy of PE [4]. 

The baseline pulse amplitude at fingertip level is highly dependent on digital blood flow and sympathetic tone, as is evidenced by a marked reduction in digital pulse amplitude after the administration of phenylephrine, an alpha-adrenergic vasoconstrictor agent [5]. Measurement of peripheral vasodilator response with a fingertip pulse amplitude tonometry (PAT) device is emerging as a useful method for assessing vascular function [6, 7]. In response to hyperemic flow, digital pulse amplitude increases, a response that has been shown to depend in part on NO synthesis [5]. Augmentation of pulse amplitude in the finger with hyperemia is a complex response to ischemia and reflects both changes in digital flow and digital microvessels dilation and is blunted by the presence of increased sympathetic tone. 

In this study, we investigated the pulse amplitude hyperemic response within the first 60 seconds in men with lifelong PE without vascular risk factors as a possible marker of sympathetic overtone and compared it with age-matched normal subjects. Then, in a randomized, double-blind, placebo-controlled crossover study, we investigated the effects of vardenafil fixed dose on reactive hyperemia index (RHI) variations and on intravaginal ejaculatory latency time (IELT). 

## 2. Methods

### 2.1. Inclusion Criteria

**Figure 1:**
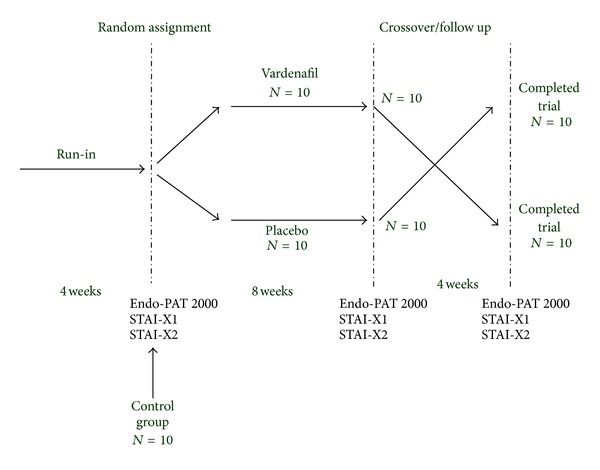
Study Design.

Vardenafil naïve men aged 18–40 years were included if they met the ESSM definition of LL-PE, as a “persistent or recurrent ejaculation with minimal sexual stimulation before, upon, or shortly after penetration and before the subject wishes it.” Patients were included if they had a score of ≥11 of the premature ejaculation diagnostic tool, a 5-item questionnaire (scored from 0 to 4 according to progressive severity of ejaculatory dysfunction) to identify men who may have a problem with ejaculating too soon during sexual activity [8]. They were entered into a 4-week run-in period, during which a diary of all sexual activity was filled. Subjects who reported at least one intercourse episode per week and IELT ≤1 minute at stopwatch in 90% of intercourse attempts during the run-in period were enrolled and randomized to receive vardenafil or placebo for 8 weeks in the double-blind, placebo-controlled crossover trial (Figure 1). IELT was defined as the time elapsed between penetration and ejaculation, and an ejaculation occurred before penetration was assigned an IELT of 0 min. Patients had to remain in a stable, single-partner relationship and have at least one sexual intercourse episode per week throughout the treatment period.

### 2.2. Exclusion Criteria

Patients were excluded if they always experienced ejaculation prior to penetration or had IELT ≥1 minute in 90% of intercourse attempts. Patients were further excluded if they had a history of ED (score of <22 on erectile function domain of the IIEF) or other ejaculatory dysfunctions to limit the possibility of a response to vardenafil as a result of treating comorbid moderate-to-severe ED. The erectile function domain of IIEF is a 5-item questionnaire (IIEF5) that investigates the presence of erectile disturbances (scored from 1 to 5 for each item) according to the capacity to obtain and maintain an erection successful for sexual intercourse along with the evaluation of confidence and satisfaction with sexual life [9]. Patients were also excluded if they used condoms or masturbated before sexual intercourse for purposes of decreasing penile sensitivity or used any other treatment for PE. Patients were excluded if they had a history of vascular disease including stroke, myocardial infarction, unstable angina, or life-threatening arrhythmias within the past 6 months or were using organic nitrates or cytochrome P450 inhibitors. Moreover, screening for hyperthyroidism (TSH, FT3, and FT4), hypogonadism (total testosterone), prolactin disorders (PRL), and prostate smears for detecting acute or subacute prostatitis were carried out before the study entry. PRL and T were measured with chemiluminescent microparticle immunoassay (CMIA, Architect System) (Abbott Laboratories, IL, USA), with detection limits of 0.05 U/L 0.07 U/L and 0.6 ng/mL and 0.28 nmol/L, respectively; intra- and interassay coefficients of variation for our laboratory were 3.5 and 4.2% at 5.8 ng/mL (PRL) and 2.3 and 3.8% at 13.7 nmol/L (T). Serum concentrations of TSH, FT3, and FT4 were determined by chemiluminescent microparticle immunoassay (CMIA, Architect System) (Abbott Laboratories, IL, USA), with limits of detection of 0.0025 mIU/L, 1.536 pmol/L, and 5.148 pmol/L, respectively.

### 2.3. Procedures

Couples were instructed to attempt sexual intercourse four or more times during the 4-week baseline period and six or more times per month during the 8-week treatment period (minimum of 24 h between doses of medication) and to record IELT for the first event after each dose in the event log by the stopwatch technique. Patients were randomly assigned within each stratum 1 : 1 to receive placebo or fixed-dose phosphodiesterase type-5 inhibitor (PDE5-i) vardenafil (10 mg) and were given 15 doses of study medication (one dose was one tablet; tablets in all groups were identical in appearance since the active principle was encapsulated) each four weeks; one dose was to be taken 30 min before anticipated sexual intercourse, and no more than one dose was allowed to be taken in a single day. Ejaculation-delaying techniques and behavioral therapy were to be avoided. Also, couples were instructed not to use condoms or topical anesthetic cream, not to pause during intercourse, or to have interrupted intromission. Furthermore, they were requested not to increase their intercourse frequency, and if intercourse took place more than once in a single session, only the first intercourse IELT was measured. Patients agreed not to change the type of treatment during the study period. Treatment efficacy was assessed at 8 weeks.

### 2.4. Psychological Evaluation

In order to identify anxiety in the psychological context of the examined subjects, the State-Trait Anxiety Inventory (STAI) test, a self-administered questionnaire, was used [10, 11]. It consists of two different scales (STAI-X1 and STAI-X2) of 20 items each, with multiple choice answers (never, sometimes, often, and always). Stai-X1 is directed at investigating the state anxiety and gives a transitory estimation of the emotional state, which varies in intensity and fluctuates in time as a function of the stressors impinging on the individual at the moment of starting the procedure. Stai-X2 is directed at relatively stable individual differences in subjects who become anxious in different circumstances [12]. Psychometric tests were performed to identify the presence of state anxiety (Stai-Xl) and trait anxiety (Stai-X2) before and after each treatment. The threshold scores for STAI questionnaires were chosen according to previously published methods [13] (normal range = 28–44 and 28–48 for the X1 and X2 form, resp.). 

### 2.5. Main Outcome Measures

The primary endpoint of the study was to evaluate differences in RHI responses between men with LL-PE and controls and to evaluate differences in RHI responses after vardenafil or placebo in men with LL-PE. In addition, differences between anxiety scores at baseline and after different treatments were evaluated. Secondary endpoint was IELT at week 8. IELT was defined as the mean duration of intercourse attempts since the last clinic visit (past 4 weeks) for which intravaginal ejaculation was reported; ejaculation occurring before penetration was assigned an IELT of 0 minutes. During the screening visit, participants and partners received instructions on the IELT measurement technique, in which partners were to activate the supplied stopwatch on vaginal penetration during sexual intercourse and to stop the stopwatch on either intravaginal ejaculation or withdrawal without ejaculation. The time noted on the stopwatch at this point was recorded as the duration of sexual intercourse until ejaculation or withdrawal. This technique has been validated elsewhere [14].

### 2.6. Determination of Peripheral Arterial Tonometry (PAT)

Each patient underwent peripheral arterial tonometry (PAT), a newly developed proprietary technology for noninvasively measuring the magnitude and dynamics of arterial tone changes in peripheral arterial beds. Digital pulse amplitude was measured, with a PAT device (Endo-PAT2000, Itamar Medical, Caesarea, Israel), in the fasting state in the supine position and both hands on the same level in a comfortable, thermoneutral environment, comprising a pneumatic plethysmograph that applies uniform pressure to the surface of the distal finger (fingers II, III, or IV) of each hand (same finger on both hands), allowing measurement of pulse volume changes in the finger. Baseline pulse amplitude was measured from each fingertip for 5 min. Arterial flow was interrupted for 5 min by a cuff placed on a proximal forearm at whichever occlusion pressure would be higher: 200 mmHg or 60 mmHg plus systolic blood pressure. Pulse amplitude was recorded electronically in both fingers and analyzed by a computerized, automated algorithm (owned by Itamar Medical) that provided the average pulse amplitude for each 30-second interval after forearm cuff deflation up to 4 min. The hyperemic response (called the PAT ratio) was expressed as the natural logarithm of the ratio of after deflation to baseline pulse amplitude in the hyperemic finger divided by the same ratio in the contralateral finger that served as control and expressed in percentage [15].

### 2.7. Statistics

We tested for differences between treatment groups by using ANCOVA. For each 30-second interval, PAT response to hyperemia is calculated as the natural logarithm transformation of PAT ratio at 90- to 120-second postdeflation time period as follows: ln[(Xh90−120/Xh120–150)/(Xc90−120/Xc120–150)]; for our study, the PAT ratio was modified, and calculated at 30- to 60-second postdeflation time period as follows: ln[(Xh30−60/Xh60–90)/(Xc30−60/Xc60–90)] with h denoting hyperemic finger, X being the pulse amplitude, and with c denoting the control finger. A multiple regression analysis was performed for STAI-X1 and STAI-X2 against the variation of PAT ratio. A *P* value < 0.05 ± SD was considered statistically significant. Statistical analysis was performed using the computer statistical package SPSS/10.0 (SPSS, Chicago, IL, USA) and SAS/6.4 (SAS Institute Cary, NC, USA).

## 3. Results

All patients were seen with their partners and interviewed about their sexual activity and patient's ejaculation function. In total, 20 patients completed the whole randomized trial study. No patient was lost at followup. There were no statistical differences in patients' characteristics at baseline at the time of randomization (Table 1) excepting for PEDT scores and prolactin levels (Table 1). 

**Figure 2:**
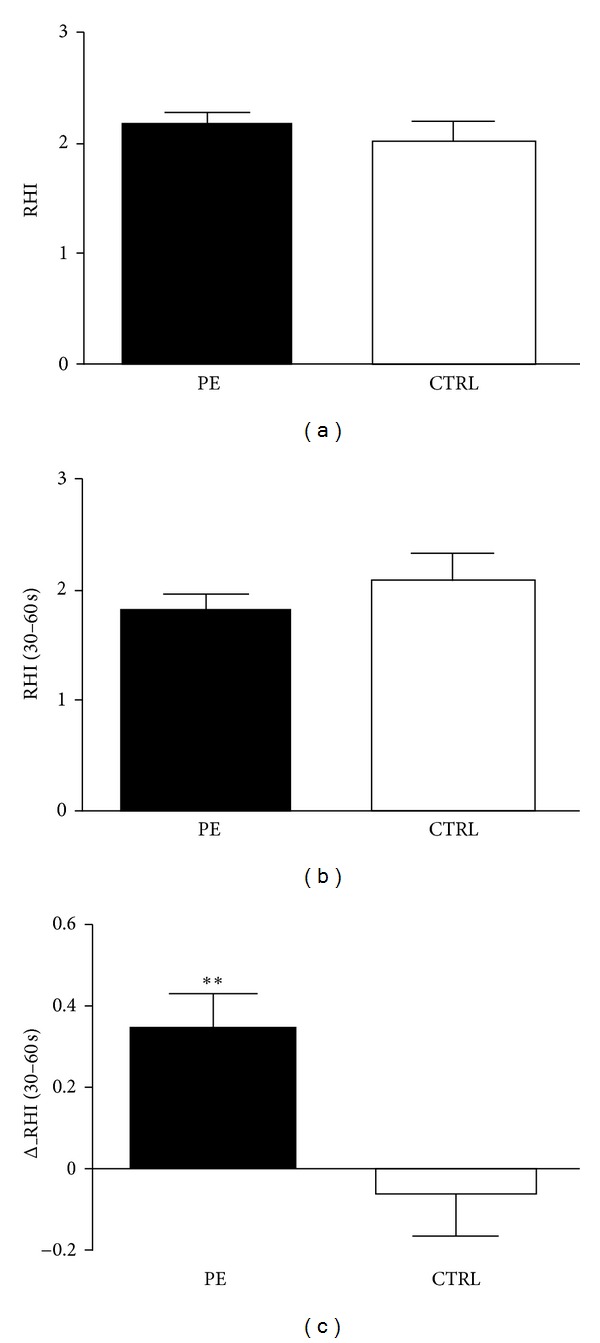
RHI values at baseline in different groups. The difference between groups is significant only when calculated as a linear variation of increment from baseline (delta RHI). PE = premature ejaculation. CTRL = controls.

At baseline, no differences in RHI values were found between groups (Figure 2(a)) when calculated as the natural logarithm transformation of PAT ratio at 90- to 120-second after deflation; when we calculate the differences in RHI, as the natural logarithm transformation of PAT ratio at 30- to 60-second after deflation (Figure 2(b)), a significant difference in the variation of RHI was found as suggested by delta_RHI calculation (*P* < 0.001, Figure 2(c)).

**Figure 3:**
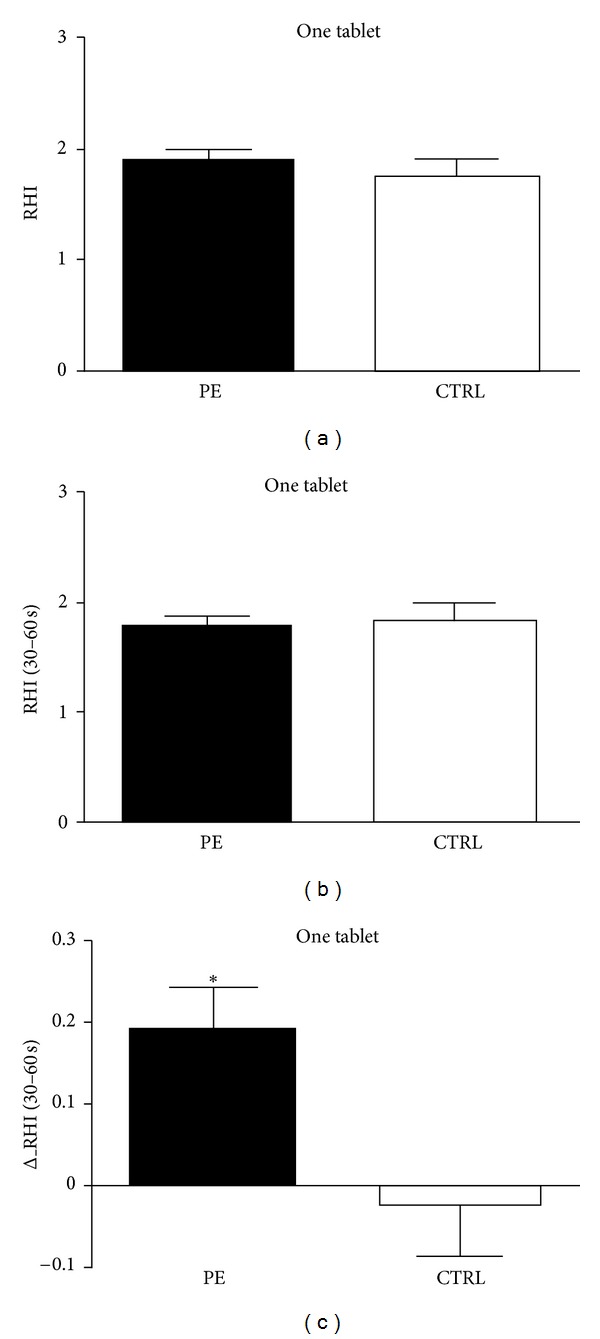
Variations in RHI values after assumption of one tablet of vardenafil 10 mg. The difference between groups is significant only when calculated as a linear variation of increment from baseline (delta RHI). PE = premature ejaculation. CTRL = controls.

**Figure 4:**
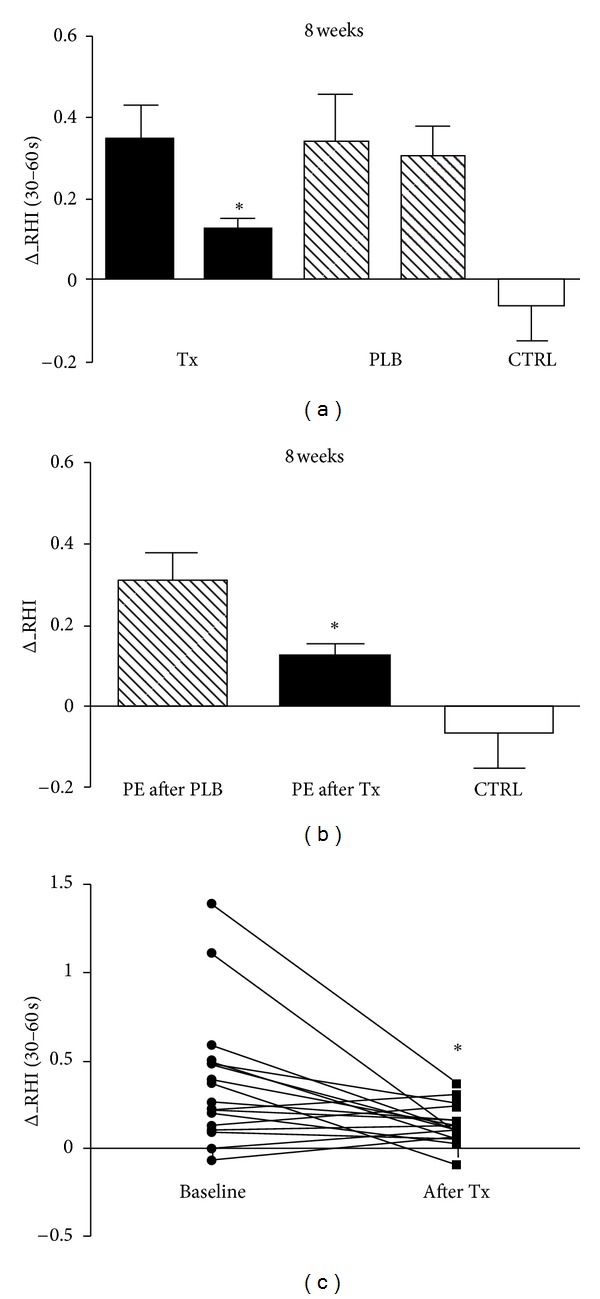
Variations in delta_RHI values after 8-week double-blind controlled trial. Tx = active treatment. PLB = placebo treatment. CTRL = controls.

All patients underwent PAT evaluation either after one tablet assumption (Figure 3) or after 8-week on-demand treatment (Figure 4). No differences in RHI values were found between groups (Figure 3(a)) when calculated as the natural logarithm transformation of PAT ratio at 90- to 120-second after deflation; when we calculate the differences in RHI, as the natural logarithm transformation of PAT ratio at 30- to 60-second after deflation (Figure 3(b)), a significant difference in the variation of RHI was found as suggested by delta_RHI calculation (*P* < 0.01, Figure 3(c)).

Also, after 8-weeks on-demand treatment, no differences in RHI values were found between groups (data not shown); when calculated as the natural logarithm transformation of PAT ratio at 30- to 60-second after deflation, a significant difference in the variation of RHI was found as suggested by delta_RHI calculation in the treatment group only (*P* < 0.01, Figure 4(a)); when delta_RHI was compared with placebo at the end of treatment, a significant difference between the two groups was found (*P* < 0.01, Figure 4(b)). Interestingly, patients with higher delta_RHI at baseline showed greater decrease after vardenafil 8-week treatment (*P* < 0.01, Figure 4(c)).

**Figure 5:**
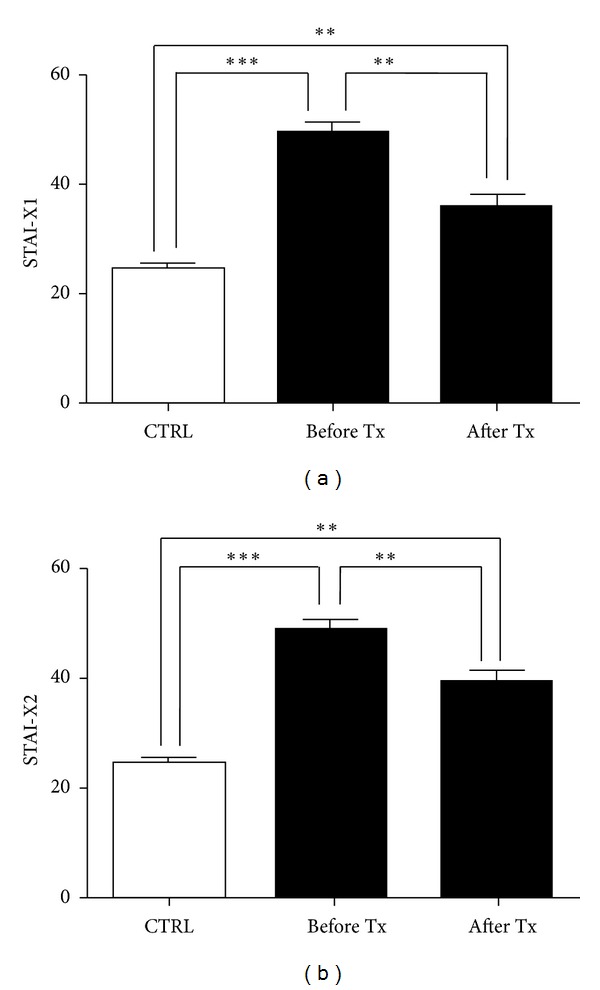
State anxiety (Stai-Xl) and trait anxiety (Stai-X2) questionnaire score changes after 8-week period treatment. Before Tx = before active treatment. Post Tx = after active treatment. CTRL = controls.

At baseline, Stai-X1 scores were different between groups (*P* < 0.0001, Figure 5(a)). Accordingly, also Stai-X2 scores showed significant differences between groups (*P* < 0.0001, Figure 5(b)). After 8 weeks of treatment with vardenafil on-demand, a significant decrease in both Stai-X1 and Stai-X2 scores were found (*P* < 0.001) which remained significant when compared with controls.

**Figure 6:**
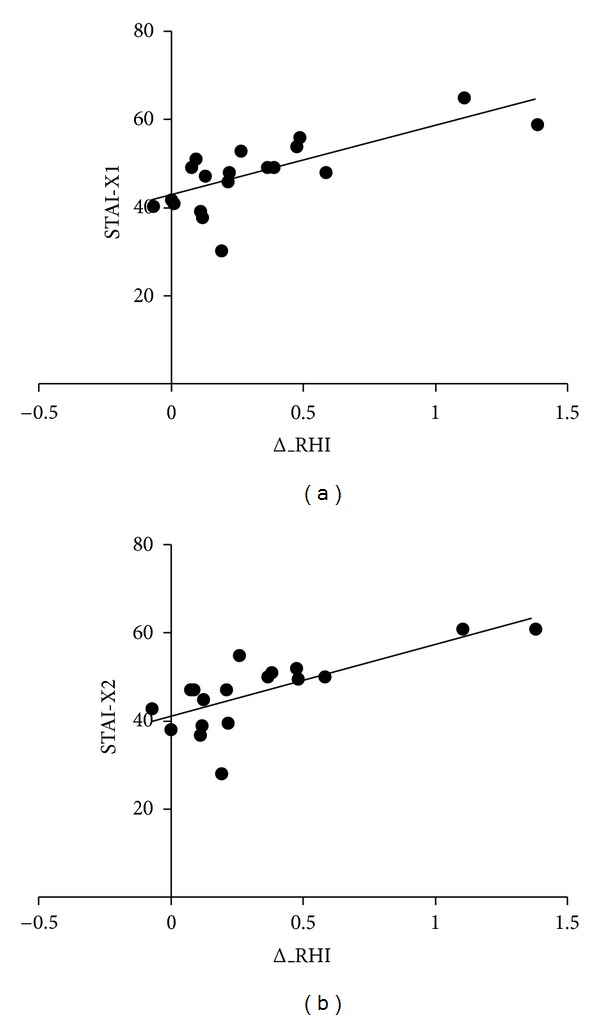
Linear ralationship between state anxiety (Stai-Xl) and trait anxiety (Stai-X2) questionnaire scores and delta_RHI variations.

Interestingly, a direct relationship between delta_RHI and Stai-X1 (*r*2 = 0.55, *P* < 0.001; Figure 6(a)) and Stai-X2 (*r*2 = 0.59, *P* < 0.001; Figure 6(b)) was found.

Baseline (geometric mean ± SD) IELT for patients randomized to vardenafil or placebo was 0.6 ± 0.3 minutes and 0.7 ± 0.3 minutes, respectively (data not shown). At the end of treatment, IELT time increased from 0.6 ± 0.3 to 4.5 ± 1.1 (*P* < 0.01, data not shown) and from 0.7 ± 0.3 to 0.9 ± 1.0 (ns), respectively. At the time of followup, patients who crossed over from placebo to vardenafil reported significant improvements in IELT (from 0.9 ± 1.0 to 2.0 ± 0.9 min, *P* < 0.05, data not shown), while on the other arm, a significant reduction in IELT was found compared with the end of study (from 4.5 ± 1.1 to 3.2 ± 1.2, *P* < 0.05, data not shown); however, this latter IELT was still superior to baseline (*P* < 0.01, data not shown). This represents a mean change per patient of 3.8 ± 1.3 minutes for patients taking vardenafil and 0.2 ± 0.3 minutes for patients taking placebo. Thus, the magnitude of the increase in IELT compared with baseline was statistically significant (*P* < 0.01, data not shown). Adverse events were significantly superior (*P* < 0.01) with vardenafil (versus placebo) after 4 weeks of treatment and were headache (10% versus 1%), flushing (12% versus 0%), and dyspepsia (10% versus 1%), which tended to disappear at the end of the study (data not shown).

## 4. Discussion

As far as we are aware, this is the first controlled study providing a possible explanation regarding the possible action of vardenafil on ejaculatory latency time. At baseline, different PAT responses were found between men with lifelong PE and controls. The finding that men with LL-PE have late vasodilator response at PAT hyperemic response is very new. In fact, when we evaluate mean RHI scores, no difference between groups was found. Indeed, a deeper evaluation of PAT response within the first 60 seconds after induced shear-stress represents a new possible application of this technique. At the outset, we demonstrate a positive relationship between PAT hyperemic response obtained within 60 seconds after vardenafil, treatment period and anxiety levels; remarkably, no PAT response to vardenafil was found in healthy controls. Interestingly, IELT improved in men treated with vardenafil while no changes were found after placebo, as previously reported in a larger study by our group [13].

Sympathetic activation causes attenuation of the PAT signal, indicative of vasoconstriction, coupled with pulse rate acceleration in addition to the typical changes reported by oximetry [16]. This technique is widely used for the diagnostic approach to sleep apneas, by giving additional information on changes of sympathetic and parasympathetic tone during sleep. Based on this knowledge, we applied this technique to men with LL-PE in order to identify differences in the autonomic control of peripheral vessels and to correlate them with the presence of anxiety. PAT software employs algorithms based on weighting two features of the PAT signal, each of which indicates sympathetic surge: amplitude attenuation (reflecting vasoconstriction) and pulse rate increase (comparable to heart rate increase) [17]. The results presented in this paper, even if obtained in a small population, are consistent with the presence of sympathetic overtone as one of the possible causes of PE in this subset of young men. The significant modification of RHI (expressed as delta RHI) implies that vardenafil treatment was able to improve vasodilatation through a nitric-oxide-(NO) mediated pathway. Given the improvements in ejaculatory function that have meaning for men with PE and their partners, the paucity of side-effects, and the fast onset of action, vardenafil may be offered as a treatment option in many men in which PE is associated with substantial psychological effects—for example, interpersonal distress, decreased self-confidence, and relationship difficulties that play a major role in the pathogenesis of the disorder. Indeed, psychological causes of PE, that is, increased performance anxiety, are well-known causes of PE and are usually treated with psychosexual therapies [18]. Furthermore, there is a real possibility for the motivated couple that the combination of pharmacotherapy with a PDE5-i and behavioral techniques may yield greater improvements in IELT.

The thoracolumbar sympathetic and the sacral parasympathetic and somatic spinal ejaculatory center (Onuf's nucleus) play a pivotal role in ejaculation because they integrate peripheral and central signals and send coordinated outputs to pelviperineal anatomic structures that allow a normal ejaculatory process to occur [19]. PDE5i may exert their influence both centrally and peripherally. The NO/cGMP pathway seems to play a role in sexual behavior via a central effect [20, 21]. Some authors have demonstrated that NO decreases central sympathetic output to the periphery via a cGMP-dependent mechanism or through interactions with other neurotransmitters. Specifically, a decrease of sympathetic tone by NO activity in the MPOA is related to inhibition of ejaculation [22]. Moreover, PDE5i may increase sexual arousal by acting in the central nervous system, in part mediated by the activation of mesolimbic dopaminergic neurons [23]. Many lines of evidence support the presence and activity of the NO/cGMP and NO/cAMP signaling pathways in the vas deferens (VD), smooth muscles, prostate, and urethra. Thus, those pathways may be responsible for the peripheral effect of PDE5i in the relaxation of penile corporal smooth muscles and could also affect smooth muscle in the VD, seminal vesicles (SVs), prostate, and urethra. A recent study demonstrated that the phasic contractile activity induced by means of electrical field stimulation of the SV tissue was most effectively inhibited by the PDE4 inhibitor rolipram and the PDE5 inhibitors sildenafil and vardenafil [24]. These observations were consistent with the findings from the studies mentioned earlier, indicating that PDE5 inhibitors can abolish the contractility of isolated human SV. Overall, these findings are in agreement with both central and peripheral actions of vardenafil on sexual behavior and on ejaculation. 

The hormonal control of ejaculation and the pathophysiology of PE are still not fully understood. Corona et al. reported that testosterone, PRL, and TSH, significantly and independently contribute to the reported IELT variation in a large population of males complaining of sexual dysfunction [25]. In particular, low PRL and high testosterone levels were associated with a higher risk of PE, even after adjusting for confounding factors that include age, body mass index, medicaments, and smoking habit. Prolactin levels have also been found to be positively correlated with reported ejaculatory latency (from severe premature ejaculation to anejaculation), after excluding men with pathological hyperprolactinaemia and adjusting for SSRI use [26]. It must be emphasized that these associations were found in a population of patients without the specific use of the stopwatch method for the quantification of IELT, as it is evaluated in the present study where PRL levels were higher in LL-PE group, but within the normal range. Additionally, we can speculate that higher PRL levels might be the result, not the cause, of the loss of ejaculatory control in our study population because of the presence of an elevated anxiety trait. 

There is suggestive evidence that men with PE are more likely to endorse questionnaire items indicating anxiety. Given that PE may be psychogenic, at least in part, and possibly related to anxiety, while part of its definition is feelings of lack of ejaculatory control, bother, and distress; there is considerable room for a placebo effect in all studies on the management of PE. In the present study, we observed an inconsistent placebo effect probably due to the design that considered an extension crossover period of time. We firstly demonstrated that PAT hyperemic response was strongest in the 30- to 60-second interval after fingertip flow was restored. The logarithmic transformation of the PAT ratio and selection of the 30- to 60-second time interval increased the overall association with the presence of a baseline constricted arterial tone, suggesting that this may be the optimal method for assessing the presence of sympathetic overactivity. The selected time period includes the portion of hyperemic response that has been previously shown to depend in part on NO production. Further studies in larger populations are needed to validate the possibility that the PAT hyperemic response is decreased in men with LL-PE and to validate the use of PAT as a diagnostic tool to identify possible responders to PDE5-I in the clinical setting.

In conclusion, fixed-dose vardenafil 10 mg is a safe and effective treatment in the absence of ED in young men with LL-PE and high anxiety scores. Vardenafil effects on IELT and PAT responses are encouraging; even if this is a pilot study, this represents indirect evidence that neurobiological patterns of PE still need to be investigated. Further larger population controlled studies to confirm the beneficial effects of vardenafil are warranted.

## Figures and Tables

**Table 1:** Demographic characteristics at baseline.

	LL-PE (*n* = 20)	CTRL (*n* = 10)
Age (ys)	31 ± 9	34 ± 9
SBP (mmHg)	115 ± 15	117 ± 17
DBP (mmHg)	79 ± 13	81 ± 14
BMI	22.5 ± 1	22 ± 0.9
AI (%)	−10 ± 14	−9 ± 15
AI@75 (%)	−16 ± 12	−16 ± 11
Cigarette smoking (%)	40	40
PEDT score	18 ± 0.8**	7 ± 0.6
IELT (minutes)	0.6 ± 0.3	11 ± 3***
TSH (mUI/mL)	2.1 ± 0.4	2.4 ± 0.3
Testosterone (ng/mL)	5.9 ± 1.4	5.8 ± 1.6
Prolactin (ng/mL)	12.2 ± 2.4	9.1 ± 2.8*

LL-PE: lifelong premature ejaculation. CTRL: controls. SBP: systolic blood pressure. DBP: diastolic blood pressure. BMI: body mass index. AI: augmentation index. AI@75: augmentation index corrected by heart rate. PEDT: premature ejaculation diagnostic tool. IELT: intravaginal ejaculatory latency time. TSH: thyroid stimulating hormone. *P < 0.05; **P < 0.01; ***P < 0.001.
